# The Prognostic Role of Tertiary Lymphoid Structures and Immune Microenvironment Signatures in Early-Stage *EGFR*-Mutant Lung Adenocarcinoma

**DOI:** 10.3390/cancers17142379

**Published:** 2025-07-17

**Authors:** Wei-Hsun Hsu, Chia-Chi Hsu, Min-Shu Hsieh, James Chih-Hsin Yang

**Affiliations:** 1 Graduate Institute of Oncology, College of Medicine, National Taiwan University, Taipei 100, Taiwan; whhsu1977@ntu.edu.tw (W.-H.H.); cchsu2@ntuh.gov.tw (C.-C.H.); 2 Department of Oncology, National Taiwan University Hospital, Taipei 100, Taiwan; 3 Department of Pathology, National Taiwan University Cancer Center, National Taiwan University Hospital, Taipei 106, Taiwan; 4 Department of Medical Oncology, National Taiwan University Cancer Center, National Taiwan University Hospital, Taipei 106, Taiwan; 5 Centers of Genomic and Precision Medicine, National Taiwan University, Taipei 100, Taiwan

**Keywords:** *EGFR*-mutant lung adenocarcinoma, tertiary lymphoid structure, tumor immune microenvironment, disease-free survival

## Abstract

Background/Objectives: The role of tertiary lymphoid structures (TLSs) in cancer prognosis is well established, yet their significance in early-stage *EGFR*-mutant lung adenocarcinoma remains unclear. While outcomes for early-stage lung cancer are generally better than those of late-stage disease, recurrence remains a significant challenge. This study investigates the prognostic value of TLSs and their molecular characteristics in early-stage *EGFR*-mutant lung adenocarcinoma. Methods: TLSs were identified in tumor samples using multiplex immunohistochemistry (IHC), and their density was quantified. The PD-L1 tumor proportion score (TPS) and TLS density were analyzed for associations with disease-free survival (DFS). Gene expression profiling was performed to compare tumor microenvironment signatures between high- and low-TLS-density groups. Results: High TLS density correlated with significantly longer DFS (43 vs. 20.5 months, *p* = 0.0082). No relationship was found between TLS density and PD-L1 TPS or *EGFR* mutation subtype. Transcriptomic analysis revealed upregulated immune response genes in the high-TLS-density group, including those involved in T and B cell activation. Low-TLS-density tumors exhibited gene signatures promoting tumor growth, such as cell cycle and WNT pathway activation. Conclusions: In summary, TLS density is a potential prognostic biomarker for DFS in early-stage *EGFR*-mutant lung adenocarcinoma, independent of PD-L1 TPS or *EGFR* mutation subtype. Enhanced immune activation in high-TLS-density tumors highlights TLSs as a potential target for improving outcomes in these patients.

## Simple Summary

In Asian populations, approximately 50–60% of non-small cell lung cancer (NSCLC) patients harbor EGFR mutations. For patients with early-stage *EGFR*-mutant lung adenocarcinoma, surgery remains the standard treatment. However, disease recurrence remains a significant concern even after curative resection. Identifying reliable prognostic biomarkers is therefore critical to improving risk stratification and guiding postoperative management. In this study, we show that high levels of tertiary lymphoid structures (TLSs) are associated with longer disease-free survival, independent of PD-L1 expression and *EGFR* mutation subtype. Our transcriptomic analysis further reveals that TLS-rich tumors have stronger immune activation, suggesting a more favorable tumor microenvironment. These findings highlight TLS density as a promising biomarker that may help guide postoperative therapeutic strategies in early-stage *EGFR*-mutant lung cancer.

## 1. Introduction

Tertiary lymphoid structures (TLSs) are ectopic lymphoid organs with an aggregative structure of immune cells, especially B and T lymphocytes [1]. The germinal center (GC) inside the TLSs show characteristics similar to those within the secondary lymphoid organs (SLOs) such as the tonsils, the spleen, Peyer’s patches, and mucosal tissues, which are responsible for proliferation, somatic hypermutation, class-switch recombination, and selection of B lymphocytes, crucial processes to protect the body against foreign pathogens [1,2]. Compared with the precise locations of SLOs, TLSs often develop at the sites of chronic inflammation and have been identified in many types of cancer [2,3]. Studies recently conducted revealed that the expression of TLSs in patients was positively correlated to the clinical outcomes in several cancer types including melanoma, renal carcinoma, and sarcomas [3,4,5,6]. However, the differences in the tumor microenvironment signature between *EGFR*-mutant lung adenocarcinoma patients with high and low TLS expression are still not fully clear. It is crucial to reveal the critical molecules and signatures of the tumor microenvironment that are associated with the expression of TLSs.

Lung cancer has been the leading cause of cancer-related death for over two decades worldwide, and mutations on the epidermal growth factor receptor (*EGFR*) are the commonest driver genes in the Asian population [7,8]. *EGFR*-mutant lung adenocarcinoma was regarded as a cancer type with “immune-cold” and “immunosuppressive” properties; this is important to consider in the pursuit to unveil insights into tumor immune microenvironment in *EGFR*-mutant lung adenocarcinoma [9,10]. Clinically, EGFR tyrosine kinase inhibitor (EGFR-TKI) therapies have been shown to be effective in late-stage (advanced) *EGFR*-mutant non-small cell lung cancer (NSCLC) patients [11,12,13,14], and the surgical removal of tumor is still the standard option for early-stage NSCLC patients [8,15]. Although the outcomes of patients with early-stage NSCLC were better than those of patients with advanced NSCLC, recurrence always inevitably occurred. Therefore, it is important to find the therapeutic biomarker for preventing recurrence and prolonging survival in early-stage *EGFR*-mutant lung adenocarcinoma patients.

In this study, we identified TLSs in early-stage *EGFR*-mutant lung adenocarcinoma patients and investigated the relationship between disease-free survival (DFS) and TLS density in these patients. Additionally, we examined the clinical association between the programmed death-ligand 1 (PD-L1) tumor proportion score (TPS) and TLSs in early-stage *EGFR*-mutant lung adenocarcinoma patients. Furthermore, we revealed the differentiated gene signatures and molecular functions between high- and low-TLS-density early-stage *EGFR*-mutant lung adenocarcinoma patients.

## 2. Results

### 2.1. Identification of Tertiary Lymphoid Structures in Early-Stage EGFR-Mutant Lung Adenocarcinoma

**Figure 1:**
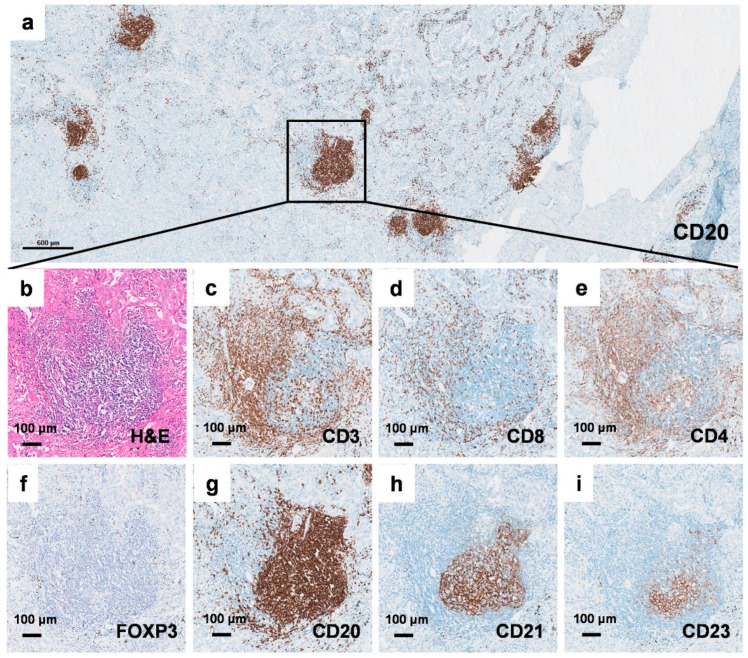
Identification of tertiary lymphoid structures in early-stage EGFR-mutant lung adenocarcinoma. (**a**) Continuous FFPE slides of early-stage EGFR-mutant lung adenocarcinoma stained with (**b**) H&E, (**c**) CD3, (**d**) CD8, (**e**) CD4, (**f**) FOXP3, (**g**) CD20, (**h**) CD21, and (**i**) CD23,. The scale bar represents 600 μm for (**a**) and 100 μm for (**b**–**i**).

**Figure 2:**
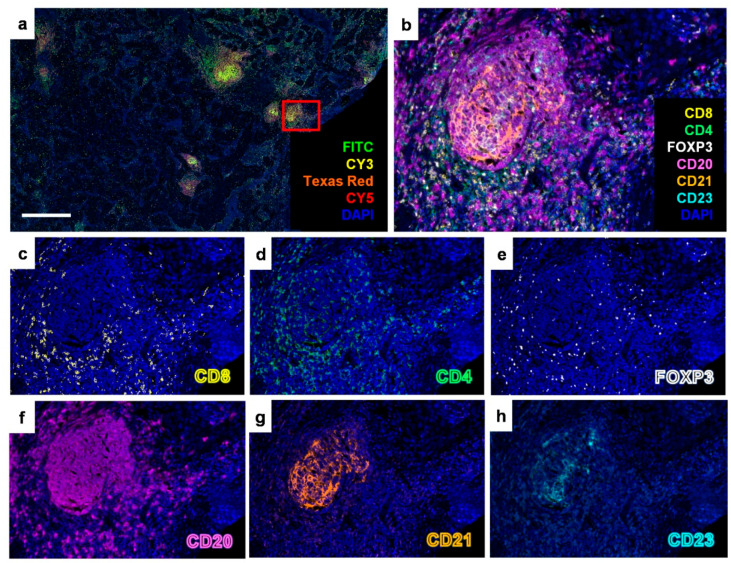
Multiplex IHC staining explored the colocalization of the markers for tertiary lymphoid structures in early-stage *EGFR*-mutant lung adenocarcinoma. (**a**) The FFPE slide stained via multiplex IHC staining (POLARIS, Medina, MI, USA). (**b**) The red box in (**a**) indicates a representative TLS, which is shown at higher magnification in (**b**) after spectral unmixing and the single-color images for (**c**) CD8, (**d**) CD4, (**e**) FOXP3, (**f**) CD20, (**g**) CD21, and (**h**) CD23 were obtained by using inForm software (version 2.4). The scale bar is 800 μm.

To validate the existence of TLSs in early-stage *EGFR*-mutant lung adenocarcinoma, we performed immunohistochemical (IHC) staining on continuous formalin-fixed and paraffin-embedded (FFPE) tumor surgical sections. The antibodies used targeted total T lymphocytes (CD3), cytotoxic T lymphocytes (CD8), helper T lymphocytes (CD4), regulatory T lymphocytes (FOXP3), B lymphocytes (CD20), and FDCs in the germinal center (either CD21 or CD23) (Figure 1). The presence of TLSs in early-stage *EGFR*-mutant lung adenocarcinoma was further confirmed using multiplex fluorescent IHC staining (Figure 2). These results demonstrate that TLSs can be identified in early-stage *EGFR*-mutant lung adenocarcinoma, despite the tumor immune microenvironment in *EGFR*-mutant lung adenocarcinoma often being characterized as an “immune-cold” and “immunosuppressive” phenotype.

### 2.2. Tertiary Lymphoid Structure Rather than PD-L1 TPS Serves as a Favorable Prognostic Factor in Early-Stage EGFR-Mutant Lung Adenocarcinoma

**Figure 3:**
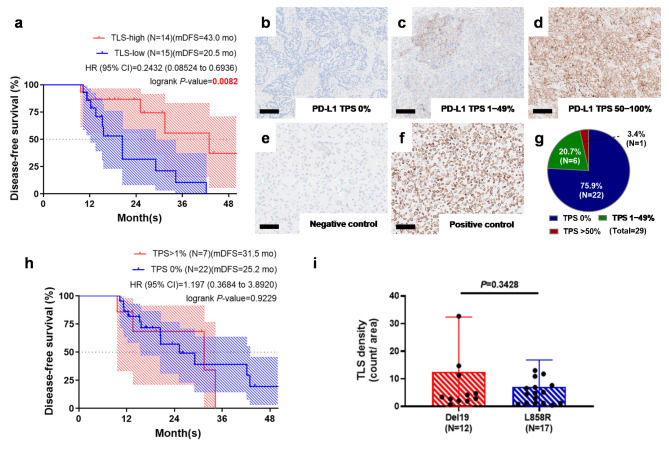
Tertiary lymphoid structures, rather than PD-L1 TPSs, determined the disease-free survival in early-stage *EGFR*-mutant lung adenocarcinoma. (**a**) The Kaplan–Meier plot representing the DFS of early-stage *EGFR*-mutant lung adenocarcinoma patients with a high or low density of TLSs; diagonal shadow bands represent 95% confidence intervals. (**b**–**g**) PD-L1 TPSs and proportion validated by using PD-L1 IHC 22C3 pharmDx. (**h**) The analyzed disease-free survival; diagonal shadow bands represent 95% confidence intervals. (**i**) A bar chart representing the difference in the densities between exon 19 deletion and L858R early-stage *EGFR*-mutant groups. TPS, tumor proportion score; DFS, disease-free survival; TLS, tertiary lymphoid structure.Scale bar: 100 μm in (**b**–**f**).

We subsequently evaluated the density of TLSs in 29 early-stage *EGFR*-mutant lung adenocarcinoma patients, as detailed in Table 1. Using the median value of TLS counts per square centimeter as a cutoff (4.5 counts/cm^2^), we categorized the patients into two groups. Notably, median disease-free survival (mDFS) was significantly longer in the high-TLS-density group at 43 months, compared to 20.5 months in the low-TLS-density group (*p* = 0.0082) (Figure 3a).

We then investigated the correlation between PD-L1 tumor proportion score (TPS) (Figure 3b–f), TLS expression, and clinical outcomes in early-stage *EGFR*-mutant lung adenocarcinoma. Of the 29 patients analyzed, 22 (75.9%) exhibited a PD-L1 TPS density of 0%, 6 (20.6%) had a TPS density ranging from 1 to 49%, and 1 (3.4%) showed a TPS density of 50% or higher (Figure 3g). Notably, there were no statistically significant differences in PD-L1 TPS density between the *EGFR* mutation groups characterized by exon 19 deletions and L858R substitutions (*p* = 0.5412; Figure S1).

We found no significant differences in median disease-free survival (mDFS) between patients with PD-L1 expression (PD-L1 TPS > 1%, 31.5 months) and those without (PD-L1 TPS = 0%, 25.2 months; *p* = 0.9229) (Figure 3h). Additionally, our analysis revealed no significant association between EGFR mutation subtype (*p* = 0.3428) and the density of tertiary lymphoid structures (TLSs) in early-stage *EGFR*-mutant lung adenocarcinoma (Figure 3i). In the univariate analysis, shorter DFS was associated with smoking history and low TLS density (Table 2). However, in multivariate analysis, TLS density remained the only independent predictor among clinical stage, smoking status, mutation subtype, and PD-L1 expression.

### 2.3. Exploration of the Differentiated Genes for the Tumor Microenvironment in Early-Stage EGFR-Mutant Lung Adenocarcinoma Patinets with TLS Expression

**Figure 4:**
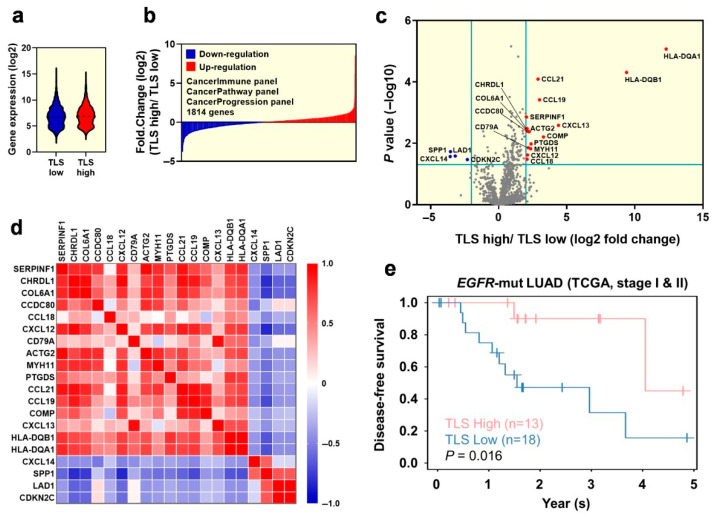
Gene signatures for the tumor microenvironment in early-stage EGFR-mutant lung adenocarcinoma with expression of TLSs. (**a**,**b**) Gene signatures examined using NanoString analysis with Cancer Immune, Cancer Pathway, and Cancer Progression panels. (**c**,**d**) A volcano plot and correlation matrix for the gene signatures with significant up- and downregulation. (**e**) Disease-free survival of early-stage EGFR-mutant lung adenocarcinoma patients from the TCGA database, stratified by high- or low-TLS-density gene signatures identified in this study. TLS, tertiary lymphoid structure; TCGA, The Cancer Genome Atlas.

We aimed to investigate the variances in the gene signatures of the tumor microenvironment between high-TLS-density and low-TLS-density early-stage *EGFR*-mutant lung adenocarcinoma. We performed nanoString analysis using the Cancer Immune, Cancer Pathway, and Cancer Progression panels. The combined results of these panels are shown in Figure 4a,b. The volcano plot identifies significantly upregulated genes (*SERPINF1*, *CHRDL1*, *COL6A1*, *CCDC80*, *CCL18*, *CXCL12*, *CD79A*, *ACTG2*, *MYH11*, *PTGDS*, *CCL21*, *CCL19*, *COMP*, *CXCL13*, *HLA-DQB1*, and *HLA-DQA1*, all marked in red) and downregulated genes (*CXCL14*, *SPP1*, *LAD1*, and *CDKN2C*, all marked in blue) in the high-TLS-density group compared to the low-TLS-density group (Figure 4c). Additionally, the correlation matrix demonstrates strong interrelations among these genes (Figure 4d).

Data from patients with lung adenocarcinoma in The Cancer Genome Atlas (TCGA) dataset were retrieved to assess the prognostic impact of the aforementioned gene set on early-stage *EGFR*-mutant lung adenocarcinoma [16]. Notably, patients with a high gene signature experienced longer disease-free survival (DFS) compared to those with a low gene signature (Figure 4e). These findings indicate that the identified gene set is associated with the presence of tertiary lymphoid structures (TLSs) and may also significantly enhance DFS in early-stage *EGFR*-mutant lung adenocarcinoma.

### 2.4. Transcriptome Analysis Reveals the Crucial Immune Cell Type and Molecular Functions Within the Tumor Microenvironment

**Figure 5:**
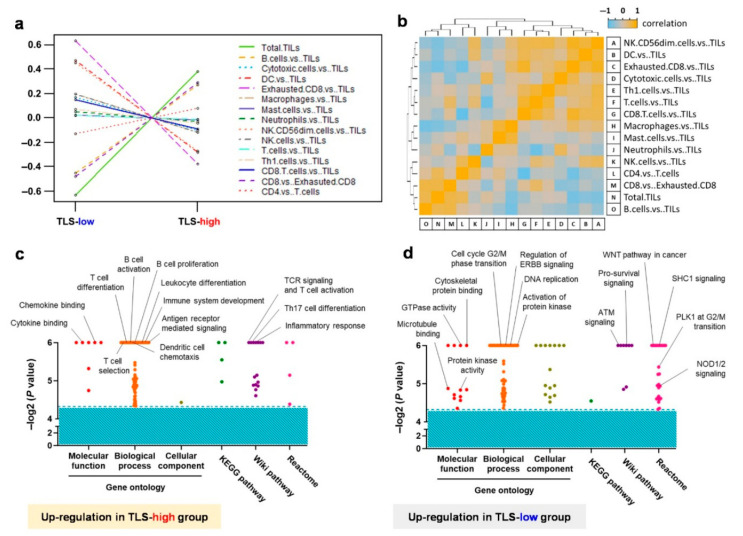
Transcriptome analysis revealing the crucial immune cell type and molecular functions within the tumor microenvironment. (**a**) Changes in immune cell types and (**b**) their correlations, as determined by NanoString analysis and nSolver software (version 4.0). Gene-set enrichment analysis revealed the molecular functions enriched in the high-TLS-density (**c**) and low-TLS-density groups (**d**). TLS, tertiary lymphoid structure.

We conducted a cell type score analysis using data obtained from NanoString. The results confirmed that the expression levels of TLS components, including B cells (indicated by a golden dashed line), CD4 T cells (shown with a red dotted line), and CD8 T cells (marked by a purple dashed line), were higher in the high-TLS-density group compared to the low-TLS-density group, as illustrated in Figure 5a,b. Gene Set Enrichment Analysis (GSEA) revealed that the high-TLS-density group exhibited enhanced functions related to immune response activation, such as T cell differentiation and activation, B cell proliferation and activation, cytokine and chemokine binding, antigen receptor-mediated signaling, and inflammatory responses, as detailed in Figure 5c. In contrast, the low-TLS-density group showed upregulation of functions supportive of tumor growth, including cell cycle transitions, DNA replication, activation of protein kinase, WNT pathway, and ATM signaling, as shown in Figure 5d. These findings elucidate the rationale behind early-stage *EGFR*-mutant lung adenocarcinoma patients with high TLS expression demonstrating longer DFS compared to those with low TLS expression.

## 3. Discussion

In the current study, we employed multiplex fluorescent IHC staining to identify the tertiary lymphoid structures (TLSs) in early-stage *EGFR*-mutant lung adenocarcinoma patients, despite this cancer type being previously characterized as “immune-cold” and “immunosuppressive”. Furthermore, we discovered that early-stage *EGFR*-mutant lung adenocarcinoma patients with high TLS expression demonstrated longer disease-free survival (DFS) compared to those with low TLS expression. We also ascertained that neither the PD-L1 tumor proportion score (TPS) nor the *EGFR* mutation subtype correlated with the expression of TLSs in early-stage *EGFR*-mutant lung adenocarcinoma patients. These findings suggest that TLS expression serves as a biomarker for longer DFS, independent of PD-L1 TPS and *EGFR* mutation subtype, in early-stage *EGFR*-mutant lung adenocarcinoma. Transcriptome analysis unveiled the differences in gene signatures between patients with high and low TLS expression, thereby providing potential candidates for pharmaceutical targeting.

The clinical impact of TLSs has been documented in several types of cancer [3]. For instance, the expression of TLSs was associated with longer disease-free survival (DFS) in patients with HER2-positive breast cancer [17]. In lung cancer, *EGFR* mutations are associated with increased PD-L1 activation, promoting immune evasion and creating an immunosuppressive tumor microenvironment (TME) by recruiting tumor-associated macrophages (TAMs) and regulatory T cells (Tregs) [18]. Lung adenocarcinomas with *EGFR* mutations exhibit reduced tertiary lymphoid structure (TLS) formation compared to those without *EGFR* mutations [19]. Elevated PD-L1 expression in tumors is also linked to lower densities of CD20+ B cells and CD4+ T cells within the TME [20]. A meta-analysis demonstrated that the presence of CD8+ cytotoxic T cells, CD20+ B cells, natural killer (NK) cells, and FOXP3+ Tregs is significantly associated with improved survival in early-stage lung cancer [21]. Retrospective analyses have further revealed that a higher density of tumor-infiltrating CD20+ B cells correlates with better responses to EGFR tyrosine kinase inhibitors (TKIs) in advanced lung cancer patients harboring *EGFR* mutations [22]. In this study, we focused specifically on early-stage *EGFR*-mutant lung adenocarcinoma and characterized the presence of TLSs within this subtype. Unlike other studies that included various lung cancer subtypes, our analysis was limited to *EGFR*-mutant cases. The majority of samples in our cohort were PD-L1-negative, aligning with real-world observations of *EGFR*-mutant lung adenocarcinoma. We found that an abundance of TLSs was associated with improved survival outcomes regardless of PD-L1 expression, consistent with previous findings that highlight the role of CD20+ B cells in the tumor microenvironment.

Reduced TIL-B presence in cases with tumor-invaded lymph nodes is linked to diminished-memory B cell differentiation, potentially due to decreased IFN-gamma signaling by NK cells [23]. A recent study highlights that a higher density of immature TLSs in the tumor microenvironment correlates with poorer clinical outcomes [24]. Additionally, in advanced lung cancer, the number and size of TLSs have been identified as biomarkers for survival and the effectiveness of immunotherapy [25]. EGFR inhibition has been shown to enhance MHC class I and II antigen presentation [26]. Consequently, in cases with low TLS density, EGFR-targeting agents may help restore TLS maturation [27]. For early-resected lung cancers with *EGFR* mutations, adjuvant EGFR TKIs could be prioritized for patients with a low TLS density in the tumor microenvironment.

Although TLS expression has been shown to be prognostic for the efficacy of immune checkpoint inhibitors (ICIs), evidence in *EGFR*-mutant lung cancer remains limited. Monotherapy with ICIs or their combination with chemotherapy agents has failed to demonstrate superior outcomes compared to chemotherapy alone [28,29]. Further investigation is needed to determine whether TLS expression could serve as a more effective biomarker for selecting patients who might benefit from ICI-containing treatments.

The limitations of our study include, firstly, the small sample size. Although the median cutoff is mentioned, TLS counts varied significantly between individuals, especially given our small sample size. This variability limits the reproducibility and clinical applicability of the method. A second limitation is the focus on surgically resected samples. As a result, our understanding of TLS status in the metastatic setting is limited. It remains unclear whether systemic treatment will have an impact on the tumor microenvironment. Additionally, the identification and quantification of TLSs remain technically challenging due to the lack of a standardized evaluation method. Tumor heterogeneity across different tissue sections may affect TLS detection, as smaller TLSs may be missed upon visual inspection, while closely clustered larger TLSs might be misinterpreted as a single structure, potentially leading to inaccurate counts. The strength of this study lies in its focus on TLS formation and the tumor microenvironment in *EGFR*-mutant lung cancer, providing valuable insights into this specific subtype of the disease. In the future, biomarker-directed prospective studies utilizing TLS density are warranted to further validate the significance of TLS density in cancer treatment.

## 4. Materials and Methods

### 4.1. Reagents and Antibodies

Opal fluorescent dyes 4′,6-Diamidino-2-Phenylindole (DAPI) and buffers for antigen retrieval were purchased from Akoya Biosciences. The antibodies against CD3, CD4, CD8, forkhead box P3 (FOXP3), CD20, CD21, and CD23 were purchased from Abcam. A delimiting pen was purchased from Dako (Carpinteria, CA, USA). ProLong™ Diamond Antifade Mountant was purchased from ThermoFisher Scientific (Waltham, MA, USA).

### 4.2. Immunohistochemical (IHC) Staining

Immunohistochemistry was performed using the VENTANA BenchMark ULTRA IHC autostainer (Roche Diagnostics, Basel, Switzerland) with antibodies specifically targeting particular immune cell types. The clones and dilutions for each antibody are listed in Table A1.

### 4.3. Multiplex Fluorescent IHC Staining and Analysis

Formalin-fixed paraffin-embedded (FFPE) slides (4 μm across) were prepared for multiplex IHC staining. Deparaffinization was carried out via the incubation of the FFPE slides at 70 °C for 4 h; then, they were washed with xylene solution twice for 20 min each, followed by hydration with an ethanol gradient (100%, 96%, and 75%) ending with distilled water. After the fixation of the tissues with a 10% neutral-buffered formalin (NBF) solution (Macron Chemicals, Center Valley, PA, USA), antigen retrieval was achieved by microwave treatment. Multiplex IHC staining was performed by using Opal Manual IHC Kit (Akoya Biosciences, Marlborough, MA, USA) in accordance with the manufacturer’s instructions. The antibodies against CD4, CD8, FOXP3, CD20, CD21 and CD23 were used to identify CD4+ T lymphocytes (helper T cells), CD8+ T lymphocytes (cytotoxic T cells), CD20+ B lymphocytes, CD4+/FOXP3+ regulatory T lymphocytes (Treg), and CD21+/CD23+ follicular dendritic cells. The DAPI was used to identify nuclei. The stained slides were scanned using Vectra Polaris Imaging System (Akoya Biosciences, Marlborough, MA, USA). TLS quantification was performed according to the procedures followed in a previous study [4]. An aggregate of B cells surrounded by T cells was considered a tertiary lymphoid structure, and these cells were counted using the stamp function (466 μm × 349 μm) of PhenoChart software (version 2.2.0). The area of stained tissue was calculated using QuPath software (version 0.5.1), and the density of tertiary lymphoid structures was then obtained.

### 4.4. PD-L1 Tumor Proportion Score (TPS) Measurement

The PD-L1 TPS was identified according to the PD-L1 expression of tumor cells. PD-L1 IHC staining was performed using PD-L1 IHC 22C3 PharmDx kit (Dako, Glostrup Kommune, Denmark). The PD-L1 TPSs were divided into three groups (PD-L1 TPS 0%, 1–49%, and 50–100%) in accordance with previous studies [30,31], and the slides with NCI-H226 and MCF-7 pellets were represented as positive and negative controls, respectively. The PD-L1 TPS density was examined in the treatment-naïve tumors of all the *EGFR*-mutant lung adenocarcinoma patients, and all these experiments were carried out by the pathologists of National Taiwan University Hospital.

### 4.5. NanoString Analysis

PanCancer Progression, PanCancer Immune Profiling, and PanCancer Pathway Panel of NanoString analysis was performed to investigate the gene signatures of the cancer microenvironment and immune constitution within the tumors. RNA (100 ng) was extracted from 10 μm thick slides of surgically resected specimens and the NanoString analysis was performed using a NanoString Sprint instrument (NanoString Technologies, Seattle, WA, USA) with a Cancer Progression Panel and PanCancer Immune Panel. The results from NanoString were normalized to housekeeper genes, and the cell type score was analyzed using the nSolver 4.0 software.

### 4.6. Statistical Analysis

A log-rank (Mantel–Cox) test was used to compare the Kaplan–Meier-estimated survival between each subgroup, and the results were considered statistically significant at a *p* value less than 0.05. The statistical significance of the gene signatures between low- and high-TLS-density early-stage *EGFR*-mutant lung adenocarcinoma patients was determined using Student’s t-tests, and the results were considered statistically significant at a *p* value less than 0.05. Cox proportional hazards analysis was conducted using the forward stepwise likelihood ratio method, incorporating baseline factors including TLS density, clinical stage, mutation subtype, smoking history, and PD-L1 expression. Statistical analyses were performed using SPSS version 30 (IBM Corp., Armonk, NY, USA).

### 4.7. Patient Enrollment and Outcome Measurements

This retrospective exploratory study of biomarkers was conducted at the National Taiwan University Hospital (NTUH). Ethical approval was obtained from the NTUH Research Ethics Committee (protocol and approval number: 201908012RINA), with the requirement for informed consent waived. The study was performed in accordance with the Declaration of Helsinki.

Patients with histologically confirmed lung adenocarcinoma who underwent surgical resection between 2010 and 2022 and who had adequate tumor tissue available for analysis were included. Tumor staging was primarily determined according to the seventh edition of the American Joint Committee on Cancer (AJCC) staging system. Data on epidermal growth factor receptor (EGFR) mutation subtypes, smoking history, and clinical outcomes were retrospectively collected through a medical chart review. Disease recurrence was defined as the emergence of new lesions identified via computed tomography imaging. Disease-free survival (DFS) was defined as the interval between the date of initial surgical resection and the date of radiologically confirmed recurrence.

## 5. Conclusions

This study highlights the critical role of tertiary lymphoid structures (TLSs) as a prognostic indicator associated with disease-free survival (DFS) in early-stage *EGFR*-mutant lung adenocarcinoma. Patients with high TLS density exhibited significantly longer DFS, irrespective of the PD-L1 tumor proportion score or *EGFR* mutation subtype. Transcriptomic analyses revealed enhanced immune activation in high-TLS-density tumors, suggesting potential avenues for therapeutic intervention. These findings underscore the importance of TLS expression in shaping the tumor immune microenvironment and support its potential use in guiding future treatment strategies and clinical research.

## Figures and Tables

**Table 1:** Patient characteristics.

		Low TLS Density (<4 Counts/cm^2^)		High TLS Density (>4 Counts/cm^2^)		*p* Value
		*n*	(%)	*n*	(%)	
Stage AJCC	I	12	80.0%	12	85.7%	0.684
	II	3	20.0%	2	14.3%	
*EGFR* mutation	Del19	5	33.3%	7	50.0%	0.362
	L858R	10	66.7%	7	50.0%	
PDL1 TPS	<1%	13	86.7%	9	64.3%	0.307
	1–49%	2	13.3%	4	28.6%	
	≥50%	0	0.0%	1	7.1%	
Smoking	No	12	80.0%	13	92.9%	0.316
	Yes	3	20.0%	1	7.1%	

TLS, tertiary lymphoid structure; TPS, tumor proportion score.

**Table 2:** Factors associated with the disease-free survival of patients with *EGFR*-mutant lung adenocarcinoma.

Characteristic	HR (95% CI)	*p* Value	Adjusted HR (95% CI)	*p* Value
Stage (I vs. II)	0.989 (0.279 to 3.526)	0.989		
*EGFR* mutation (Del19 vs. L858R)	1.569 (0.556 to 4.427)	0.395		
Smoking history (No vs. Yes)	0.199 (0.047 to 0.846)	**0.029**	0.203 (0.038 to 1.076)	0.061
TLS (high vs. low density)	0.235 (0.074 to 0.746)	**0.014**	0.235 (0.071 to 0.774)	**0.016**
PDL1 TPS (0% vs. ≥1%)	0.823 (0.256 to 2.647)	0.744		

TLS, tertiary lymphoid structure; TPS, tumor proportion score. Bold values indicate statistical significance (*p* < 0.05).

## Data Availability

Data are contained within the article and its Supplementary Materials. Additional datasets (e.g., raw imaging data) are available from the corresponding author upon reasonable request.

## Supplementary Materials

The following supporting information can be downloaded at https://www.mdpi.com/article/10.3390/cancers17142379/s1: Figure S1: There is no difference in the PD-L1 TPS between exon 19 deletion and L858R early-stage EGFR-mutant groups.

## Appendix A

**Table A1:** Information on the antibodies used in this study.

Antibody	Species	Reference	Clone	Source	Antigen Retrieval	Concentration Dilution
CD3	Rabbit	ab16669	SP7	Abcam	pH6	1:150
CD8	Rabbit	ab178089	SP239	Abcam	pH9	1:100
CD4	Rabbit	ab213215	SP35	Abcam	pH9	1:50
FOXP3	Mouse	ab20034	236A/E7	Abcam	pH6	1:200
CD20	Mouse	ab9475	L26	Abcam	pH6	1:100
CD21	Rabbit	ab75985	EP3093	Abcam	pH6	1:300
CD23	Rabbit	ab16702	SP23	Abcam	pH6	1:100
PanCK	Mouse	ab7753	C-11	Abcam	pH6	1:200
