# Supplementary material for: The Prognostic Role of Tertiary Lymphoid Structures and Immune Microenvironment Signatures in Early-Stage EGFR-Mutant Lung Adenocarcinoma
Source: Cancers (Basel). 2025 Jul 17;17(14):2379. doi: 10.3390/cancers17142379 (PMC12293609; doi:10.3390/cancers17142379)
Supplement: Supplementary file 1 [file cancers-17-02379-s001.zip › cancers-3744990-supplementary.pdf]

Supplementary Materials

The Prognostic Role of Tertiary Lymphoid Structures and Immune Microenvironment Signatures in Early-Stage *EGFR*-Mutant Lung Adenocarcinoma

Wei-Hsun Hsu [1,2,†], Chia-Chi Hsu [1,2,†], Min-Shu Hsieh [3] and James Chih-Hsin Yang [1,2,4,5,*]

[1] Graduate Institute of Oncology, College of Medicine, National Taiwan University, Taipei 100, Taiwan; whhsu1977@ntu.edu.tw (W.-H.H.); cchsu2@ntuh.gov.tw (C.-C.H.)

[2] Department of Oncology, National Taiwan University Hospital, Taipei 100, Taiwan

[3] Department of Pathology, National Taiwan University Cancer Center, National Taiwan University Hospital, Taipei 106, Taiwan; a02035@ntucc.gov.tw

[4] Department of Medical Oncology, National Taiwan University Cancer Center, National Taiwan University Hospital, Taipei 106, Taiwan

[5] Centers of Genomic and Precision Medicine, National Taiwan University, Taipei 100, Taiwan

* Correspondence: chihyang@ntu.edu.tw; Tel.: +886-2-2322-0322 (ext. 38661); Fax: +886-2-3322-9608

† These authors contributed equally to this work.

Supplemental Figure S1 and legends

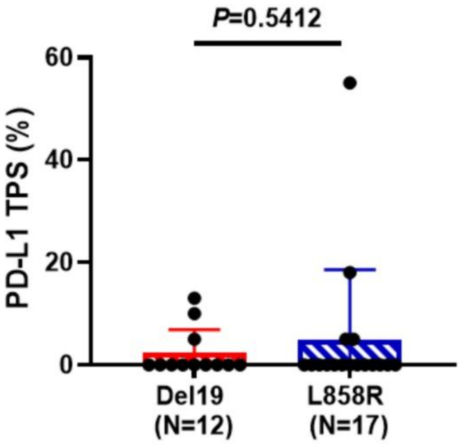

Figure S1. Comparison of PD-L1 tumor proportion score (TPS) between Del19 and L858R subtypes of early-stage *EGFR*-mutant lung adenocarcinoma. The PD-L1 TPS was evaluated by immunohistochemistry using the 22C3 pharmDx assay. No statistically significant difference was observed between the two groups (P = 0.5412, unpaired t-test). Data are presented as mean ± standard deviation.
